# Bioinformatics-based screening of key genes for transformation of tyrosine kinase inhibitor-resistant lung adenocarcinoma to small cell lung cancer

**DOI:** 10.3389/fmed.2023.1203461

**Published:** 2023-07-31

**Authors:** Ying Zhang, Qiang Chen, Ting Huang, Di Zhu, Yuanzhi Lu

**Affiliations:** ^1^Department of Oncology, First Affiliated Hospital of Jinan University, Guangzhou, China; ^2^Department of Clinical Pathology, First Affiliated Hospital of Jinan University, Guangzhou, China

**Keywords:** bioinformatic analysis, SCLC, LUAD, transformation, EGFR

## Abstract

**Purpose:**

Lung adenocarcinoma (LUAD) is a common type of lung cancer. Cancer in a small number of patients with EGFR mutations will transform from LUAD to small cell lung cancer (SCLC) during epidermal growth factor receptor tyrosine kinase inhibitor (EGFR-TKI) therapiesr. The purpose of the present study was to identify the core genes related to the transformation of LUAD into SCLC and to explore the associated molecular mechanisms.

**Methods:**

GSE29016, GSE1037, GSE6044 and GSE40275 mRNA microarray datasets from Gene Expression Omnibus (GEO) were analyzed to obtain differentially expressed genes (DEGs) between LUAD and SCLC tissues, and the results were used for network analysis of protein–protein interactions (PPIs). After identifying the hub gene by STRING and Cytoscape platform, we explored the relationship between hub genes and the occurrence and development of SCLC. Finally, the obtained hub genes were validated in treated LUAD cells.

**Results:**

A total of 41 DEGs were obtained, four hub genes (*EZH2*, *NUSAP1*, *TTK* and *UBE2C*) were identified, and related prognostic information was obtained. The coexpressed genes of the hub gene set were further screened, and the analysis identified many genes related to the cell cycle. Subsequently, LUAD cell models with TP53 and RB1 inactivation and overexpression of ASCL1 were constructed, and then the expression of hub genes was detected, the results showed that the four hub genes were all elevated in the established cell model.

**Conclusion:**

*EZH2*, *NUSAP1*, *TTK* and *UBE2C* may affect the transformation of LUAD to SCLC and represent new candidate molecular markers for the occurrence and development of SCLC.

## 1. Introduction

Lung cancer is the most common and deadly malignant tumor in the world (1) and includes two main histological subtypes: non-small cell lung cancer (NSCLC) and SCLC. SCLC accounts for approximately 20% of all lung cancer cases. It is characterized by a high growth rate, rapid doubling time, and early establishment of extensive metastatic lesions. Without treatment, SCLC can quickly lead to death (2). In clinical practice, most lung cancers are NSCLC, of which LUAD is the most common subtype. In the past, depending on the patient’s tumor stage and general health, surgery, radiation, and/or chemotherapy were the primary therapeutic methods used in the management of NSCLC (3). However, the majority of patients are discovered in the advanced malignance, which affects the patient survival rate (4). The clinical management of NSCLC has been completely transformed by advances in sequencing technologies, knowledge of the molecular changes causing tumor progression, and in-depth knowledge of anti-tumor immune mechanisms. The development of molecularly targeted therapies and immunotherapy has increased the number of available treatments and improved patient safety and survival. Notably, almost two-thirds of individuals with NSCLC contain oncogenic driver mutations, and around half of them have therapeutically targeted lesions (5, 6).Common genetic changes include mutations in *TP53*, *EGFR and KRAS*, as well as fusions of *EML4-ALK* and *ROS1* (7). However, approximately 15% of the patients experienced a histological transformation from LUAD to SCLC during EGFR-TKI treatment and gained resistance to TKIs (8). The clinical manifestations, treatment and prognosis of transformed SCLC are similar to those of primary SCLC (9). At present, the mechanism of the transformation of LUAD to SCLC is unclear. There is no molecular marker to distinguish the continuous effective treatment state of LUAD from the state before LUAD transformation. Early diagnosis and the transformation of treatment may improve the survival rate of these patients. Therefore, it is of great significance to understand the molecular mechanisms of LUAD development into SCLC, explore the molecular characteristics of SCLC transformation, development, and prognosis. These studies will provide new information to develop strategies for effective prevention, early diagnosis, and treatment of SCLC transformation.

Microarray and bioinformatics approaches have been widely used to screen genetic changes at the genomic and transcriptomic level. Herein, we analyzed four mRNA microarray datasets from the Gene Expression Omnibus (GEO) to obtain differentially expressed genes (DEGs) between SCLC and LUAD tissues. Subsequently, protein–protein interaction (PPI) network analysis was carried out to explore the relationship between different genes. The CytoHubba plug-in was used to screen hub genes, and Kaplan–Meier plotter was used to analyze the relationship between hub genes and prognosis. Combined with the analysis of the ONCOMINE database, *EZH2*, *NUSAP1*, *TTK*, and *UBE2C* were identified as core hub gene sets related to SCLC transformation. cBioPortal was used to identify genes that were closely related to changes in the core hub gene set. The clusterProfiler package of the R project was then used to perform GO enrichment analysis. Finally, the RNA and protein levels of the obtained hub genes were verified in treated LUAD cells. This discovery provides new candidate molecular markers for studying the transformation of SCLC and further explores the mechanism of transformation.

## 2. Materials and methods

### 2.1. Microarray data

The GEO database^1^ was used to obtain expression data for identifying DEGs. We selected patients with LUAD and SCLC from the GSE29016 series (10) (Illumina HumanHT-12V3.0 expression beadchip), GSE1037 series (11, 12) (CHUGAI 41K), GSE6044 series (13) (Affymetrix Human HG-Focus Target Array) and GSE40275 series (14) (Human Exon 1.0 ST Array). The basic clinical information of the selected patients is shown in Supplementary file 1: Table S1. According to the annotation information of each platform, probes were converted into the corresponding gene symbol. The GSE29016 series contained 7 SCLC tissue samples and 38 LUAD tissue samples, the GSE1037 series contained 15 SCLC tissue samples and 12 LUAD tissue samples, the GSE6044 series contained 9 SCLC tissue samples and 10 LUAD tissue samples, and the GSE40275 series contained 15 SCLC tissue samples and 8 LUAD tissue samples.

### 2.2. Identification of DEGs

GEO2R^2^ was used to identify DEGs between SCLC and LUAD samples. GEO2R is an interactive network tool that allows users to compare two or more datasets in the GEO series to identify DEGs under different experimental conditions (15). One probe set corresponding to multiple genes and genes without gene symbols were removed. Genes with multiple probe sets were averaged and retained. A fold change >2 and adjusted *p*-value <0.05 were considered statistically significant thresholds. To identify important DEGs, the Venn online tool^3^ was used to draw a Venn diagram, and overlapping DEGs were retained for further analysis.

### 2.3. Construction and module analysis of The PPI network

The STRING online database (http://string-db.org Version 11.5) (16) was used to construct the PPI network. The functional interactions between proteins was used to explore the mechanisms related to the occurrence and development of diseases. The molecular compound detection plug-in CytoHubba in Cytoscape (17) was used to cluster the generated network to reveal tightly connected regions. The most important modules in the PPI network were identified by CytoHubba, and we selected the top 4 nodes ranked by the maximal clique centrality (MCC) method.

### 2.4. Hub gene analysis

The correlation between the hub genes from TCGA was analyzed using cBioPortal^4^ (18). The online database Garber lung tumor dataset (19) from Oncomine^5^ (20) was used to analyze the expression of hub genes in normal, large cell lung carcinoma, LUAD, SCLC and squamous cell lung cancer tissue samples. Subsequently, overall survival related to hub genes was analyzed using the Kaplan–Meier curve feature of Kaplan–Meier plotter, which includes 719 patients (21). cBioPortal further screened the DEGs closely related to the hub genes. The clusterProfiler package (22, 23) of the R project was used to perform GO enrichment analysis on these genes.

### 2.5. Cell culture

293T, NCI-H1299 and NCI-H1975 cells (human lung adenocarcinoma cell line) were purchased from the Cell Bank of the Chinese Academy of Sciences (Shanghai, China). The medium required for the NCI-H1975 and NCI-H1299 cell line was RPMI 1640 medium (Gibco, Grand Island, NY, United States) supplemented with 10% fetal bovine serum (FBS, Gemini, Woodland, CA, United States) and 1% penicillin and streptomycin (Gibco, Grand Island, NY, United States). Cell lines were cultured in a 37°C incubator with 5% CO_2_.

### 2.6. Design of RB1 shRNA and production of lentivirus

To construct the RB1 knockdown lentiviral vector, a synthetic human shRNA interference sequence was first identified, and then the corresponding oligonucleotides were synthesized, annealed, and inserted into the lentiviral vector pLK0.1 AgeI and EcoRI restriction sites (both enzymes were obtained from NEB, Ipswich, MA, United States). Finally, the successful construction of the RB1-shRNA lentiviral vector was verified by sequencing. The RB1-shRNA primer sequence was as follows:


$$\begin{array}{l} RB1-\mathrm{shRNA}-F:\mathrm{CCGGCGAAATTGGATCACAGCGATACTCG}\\ \;\mathrm{AGTATCGCTGTGATCCAATTTCGTTTTTG} \end{array}$$

$$\begin{array}{l} RB1-\mathrm{shRNA}-R:\mathrm{AATTCAAAAACGAAATTGGATCACAGCG}\\ \;\mathrm{ATACTCGAGTATCGCTGTGATCCAATTTCG} \end{array}$$

### 2.7. Infections of human LUAD cells with lentivirus

Together with recombinant plasmids, psPAX and pMD2G plasmids, recombinant lentiviral vectors were transfected into 293T cells for 2 days. After filtering the lentivirus, the viral supernatant was used to infect H1299 cells and H1975 cells for 6 h. Cells were then treated with puromycin for 2 weeks. After an additional 2–5 days of culture, protein levels were checked by western blotting.

### 2.8. Overexpression lentiviral transfection

The CV237 vector, which contains the BamHI/NheI cloning site, was utilized for this experiment. Lentiviruses LV-ASCL1, designed to overexpress ASCL1, and CON524, serving as a negative control, were obtained from Shanghai Genechem Co., Ltd.. H1975 cells were transfected with LV-ASCL1 or CON524 for 16 h, and then cultured with complete medium. After 72 h of virus infection, Hygromycin B was used to screen for the target cells, with unsuccessful infections eliminated. The screening process using Hygromycin B lasted for approximately 14 days. Finally, the expression of ASCL1 was assessed using quantitative real time polymerase chain reaction (qPCR) and WB techniques.

### 2.9. Quantitative real time polymerase chain reaction

RNA was extracted using the RNA-Quick Purification Kit (Esunbio, RN001), and the concentration of the RNA was measured using Nanodrop. To remove genomic DNA, cDNA was synthesized using the PrimeScript^™^ RT Reagent Kit with gDNA Eraser (TaKaRa, RR047A). The reverse transcription was performed using the TB Green^®^ Premix Ex Taq^™^ II (TaKaRa, RR820A). The primers were designed according to the purpose of the project and synthesized by Shanghai Sangon Bioengineering Technology Service Co., Ltd.. The primer sequences are as follows:

**Table:** 

Primer name	Sequence (5′to 3′)
ASCL1-F	TCAAGTTGGTCAACCTGGGCTTTG
ASCL1-R	CGCAGTGTCTCCACCTTACTCATC
EZH2-F	GTGATAGGGAAGCAGGGACTGAAAC
EZH2-R	CAGCACCACTCCACTCCACATTC
NUSAP1-F	TGAGCATAAGCGTTCACTGACCAAG
NUSAP1-R	GAGTCTGCGTTGCCTCAGTTGTC
TTK-F	ACTTTCCACCTGCTTGTCAGTTGTC
TTK-R	GCTTGAACCTCCACTTCCTATCTGC
UBE2C-F	TGCCAGAACCCAACATTGATAGTCC
UBE2C-R	GGCTGGTGACCTGCTTTGAGTAG
β-actin-F	CTGGCACCACACCTTCTACAATGAG
β-actin-R	GATAGCACAGCCTGGATAGCAACG

### 2.10. Western blot

Cells were washed with PBS buffer and lysed with lysis buffer to extract total protein, which was quantified with a BCA Protein Assay Kit (BCA Protein Assay Kit, Thermo Fisher Scientific, USA). For western blot (WB) analysis, 30–40 μg protein samples were mixed with loading buffer. Cell lysates were then separated by SDS–PAGE electrophoresis and transferred to PVDF membranes (Merck Millipore, Darmstadt, Germany). The membrane was blocked with 5% milk in TBST buffer for 1 h and then incubated with the primary antibody overnight at 4°C. The primary antibodies used here are as follows: mouse anti-RB, Cell Signaling Technology, 9,309 (1:2000), rabbit anti-EZH2, Cell Signaling Technology, 5,246 (1:1000), rabbit anti-TTK, Cell Signaling Technology, 3,255 (1:1000), rabbit anti-NUSAP1, Proteintech, 12,024-1-AP (1:1000), rabbit anti-UBE2C, Proteintech, 12,134-2-AP (1:1000), rabbit anti-GAPDH, Cell Signaling Technology, 2,118 (1:4000), rabbit anti-ASCL1, Bioss, bs-1155R (1:1500). After 3 washes with TBST buffer, specific HRP-conjugated secondary antibodies from Jackson ImmunoResearch were added, and immunoreactivity was detected with the ECL-Plus kit (Thermo Fisher Scientific, United States).

### 2.11. Immunocytochemistry

An appropriate amount of H1975 cells in the growth phase was seeded on a 12-well plate with cell slides. When the cell confluence was 70%–80%, it was fixed with 4% paraformaldehyde at room temperature, and then permeabilized with 0.2% TritonX-100. Used 5% bovine serum albumin (BSA) to block, and added an appropriate amount of diluted primary antibody at 4°C overnight. Then added an appropriate amount of secondary antibody to the sample for incubation. To visualize staining, cells were incubated in the dark with diaminobenzidine (DAB) solution and finally counterstained with hematoxylin staining solution. After the slides were air-dried and sealed, they could be examined under a microscope.

### 2.12. Cytotoxicity test

LUAD cells were taken in the logarithmic growth phase, adjusted the cell concentration to 50 cells/μL after digestion with trypsin, we took 96-well plates to mark the blank group, control group and experimental group, each group had 3–5 duplicate wells, added 100 μL cell suspension to each well, and added 150 μL PBS to each well around. Then the 96-well plate was placed in a 37°C, 5% CO_2_ incubator for 24 h. The drug was diluted with complete medium according to a certain gradient, added to the 96-well plate, and plate was placed in the incubator for 72 h. We removed the supernatant after 72 h, prepared CCK8 working solution in a 10% ratio in the dark, mixed it and added it to the 96-well plate, placed it in a 37°C incubator for 30 min–2 h, and measured the absorbance at a wavelength of 450 nm in a microplate reader. Cell viability and IC50 was calculated based on absorbance.

### 2.13. Sequencing analysis of gene expression profiles in H1975 cells

NCI-H1975 cells infected with lentivirus expressing RB1-shRNA (*n* = 3) or pLKO.1-shRNA (*n* = 3) were cultured, and total RNA was extracted using TRIzol reagent. RNA degradation and purity were checked using 1% agarose gels, Thermo scientific NanoDrop and Agilent 2100 bioanalyzer. For gene expression analysis, gene expression profiles in H1975 cells infected with lentivirus expressing RB1-shRNA (*n* = 3) and pLKO.1-shRNA (*n* = 3) were analyzed using an Illumina HiSeq 1000 in Shanghai GeneChem Co., Ltd., (China). Raw data was analyzed using Illumina, and was obtained by removing reads containing adapter, reads containing ploy-N and low quality reads from raw data. RNA libraries were constructed from ≥1 μg of total RNA. The kit used to build the library was Illumina’s NEBNext^®^ UltraTM RNA Library Prep Kit. DESeq2 software was used to analyze differentially expressed genes (DEGs) using a model based on the negative binomial distribution, and the DEGs screening criteria were set to *p*_adj_ <0.05. Supplementary Figure S1 also includes more detailed instructions.

### 2.14. Label-free quantitative proteomics

The RB1-knockdown H1975 experimental group cells (*n* = 3) and control cells (*n* = 3) were subjected to Label-free analysis in Shanghai Genechem Co., Ltd.. Protein was extracted, and 20 μg of protein was taken from each sample for SDS-PAGE electrophoresis (constant voltage 250 V, 40 min). Subsequently, DTT was added to each sample to a final concentration of 100 mM, followed by incubation in a boiling water bath for 5 min, and cooled to room temperature. Next, 200 μL of UA buffer was added to each sample and mixed well. The mixture was then transferred to a 30kD ultrafiltration centrifuge tube for centrifugation, and the filtrate was removed. After that, 100 μL of IAA buffer solution was added, and the samples were shaken for 1 min, followed by incubation at room temperature for 30 min in the dark, and then centrifuged. Next, 100 μL of UA buffer was added, and centrifugation was performed. Then, 100 μL of 50 mM NH_4_HCO_3_ solution was added, followed by centrifugation. The collection tube was replaced, and 40 μL of trypsin buffer (4 μg trypsin in 40 μL 50 mM NH_4_HCO_3_ solution) was added. The samples were shaken for 1 min and placed at 37°C for 16–18 h. After that, the samples were centrifuged, and 40 μL of 50 mM NH_4_HCO_3_ solution was added again, followed by centrifugation, and the filtrate was collected. Finally, the peptides were desalted, lyophilized, and then reconstituted by adding 40 μL of 0.1% formic acid solution. In the Easy nLC system, 0.1% formic acid aqueous solution (solution A) and 0.1% formic acid acetonitrile aqueous solution (solution B) were used as flow phases, and the separation of peptides was performed in the chromatographic column.

The sample underwent mass spectrometry analysis using an Orbitrap Exploris 480 mass spectrometer, with a detection time of 90 min and a positive ion mode. The precursor ion scanning range was set to 350-1,200 m/z, and the resolution of the primary mass spectrometer was 120,000, with an AGC target of 300% and a primary maximum IT of 50 milliseconds. Peptides and peptide fragments were collected using the cycle time method of data dependent mode, with a cycle time of 1.5 s, an MS2 activation type of HCD, and an Isolation window of 1.6 *m*/*z*. The resolution of the secondary mass spectrometer was set to 15,000, with microscans of 1, an AGC target of 75%, and a secondary maximum IT of 35 milliseconds. The ion dynamic exclusion time was 30 s, and the normalized collision energy was set to 33%.

For data analysis, a label-free quantitative approach based on MS1 data integration was employed, and the resulting data were processed using MaxQuant software.

### 2.15. Statistical analysis

GraphPad 7 was used for data analysis, and all experiments were performed in triplicate. Data are shown as the mean ± SEM of three independent experiments. Student’s two-tailed t test was selected for statistical analysis, and *p* < 0.05 was considered statistically significant.

## 3. Results

### 3.1. Identification and analysis of DEGs in SCLC tissues

**Figure 1:**
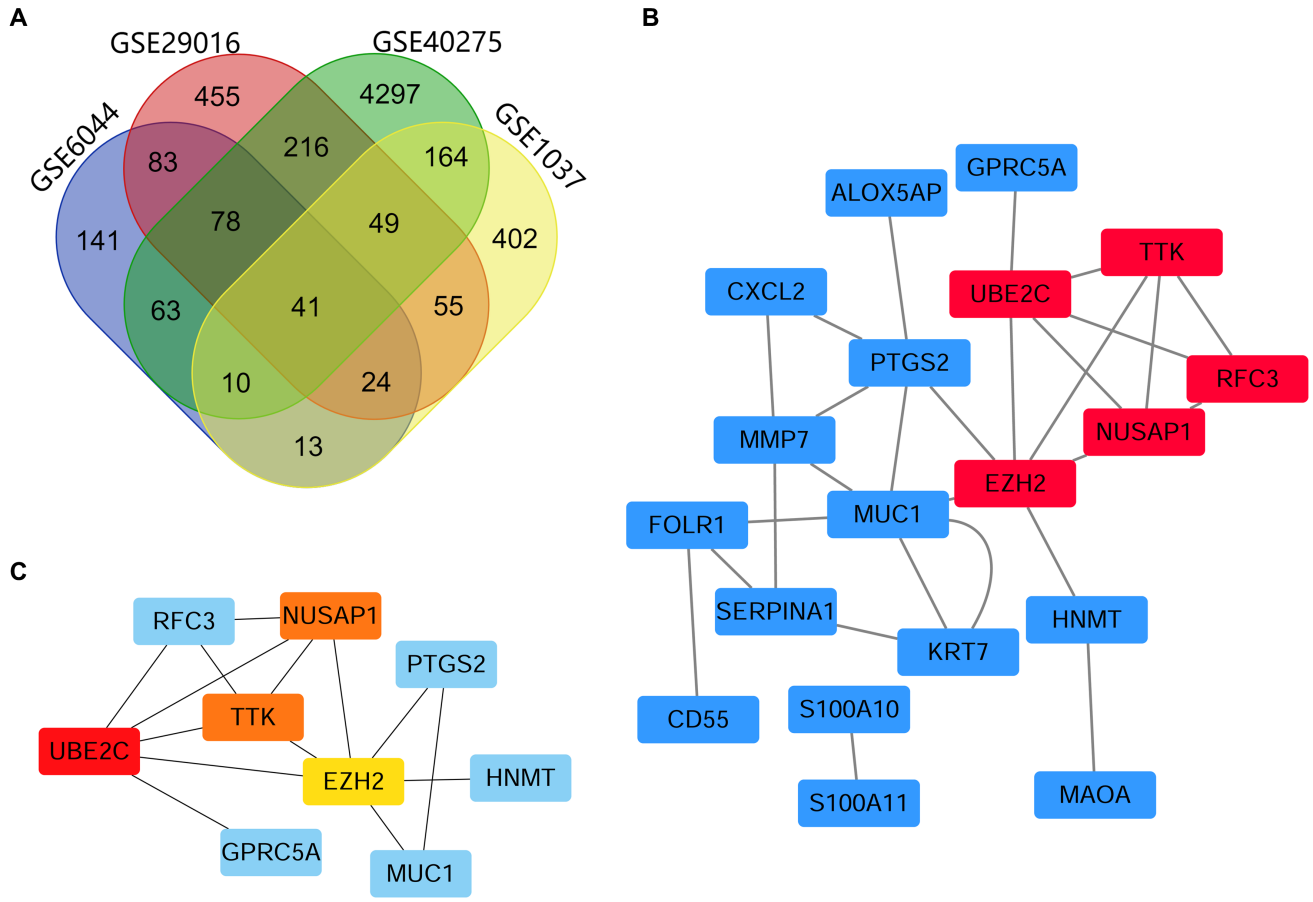
Venn map and PPI network analyses, showing the most significant module related to DEGs. **(A)** DEGs were identified from the GSE29016, GSE1037, GSE6044 and GSE40275 gene expression profiling datasets based on fold change >2 and adjusted *p*-value <0.05. The four datasets shared 41 overlapping DEGs. **(B)** PPI network constructed using 41 DEGs. Up- and downregulated genes are marked in red and blue, respectively. **(C)** The first 4 hub genes calculated using the MCC algorithm were *UBE2C*, *NUSAP1*, *TTK*, and *EZH2*. The higher the calculated score, the more critical the gene selected, and the darker the color of the network graph (where *NUSAP1* and *TTK* scores are consistent).

DEGs identified in the four microarray datasets (1,001 in GSE29016, 758 in GSE1037, 453 in GSE6044 and 4,918 in GSE40275) were screened after the chip results were normalized. As shown in the Venn diagram, 41 genes overlapped in the four datasets (Figure 1A; Supplementary file 2: Table S2), including 13 and 26 that were upregulated and downregulated, and the other two genes *DUSP6* and *RHOB* had opposite results in different datasets. The STRING database was used for screening, PPI network construction was performed, and visualization was carried out using Cytoscape (Figure 1B). Since *DUSP6* and *RHOB* belonged to the disconnected nodes in the PPI network, they were removed from the visualization. The PPI network was constructed, and significant modules were identified, with 54 edges and 19 nodes in the PPI network. The MCC algorithm of the CytoHubba plug-in was used to screen hub genes and identify the most closely connected modules (Figure 1C).

### 3.2. Selection and analysis of hub genes

**Figure 2:**
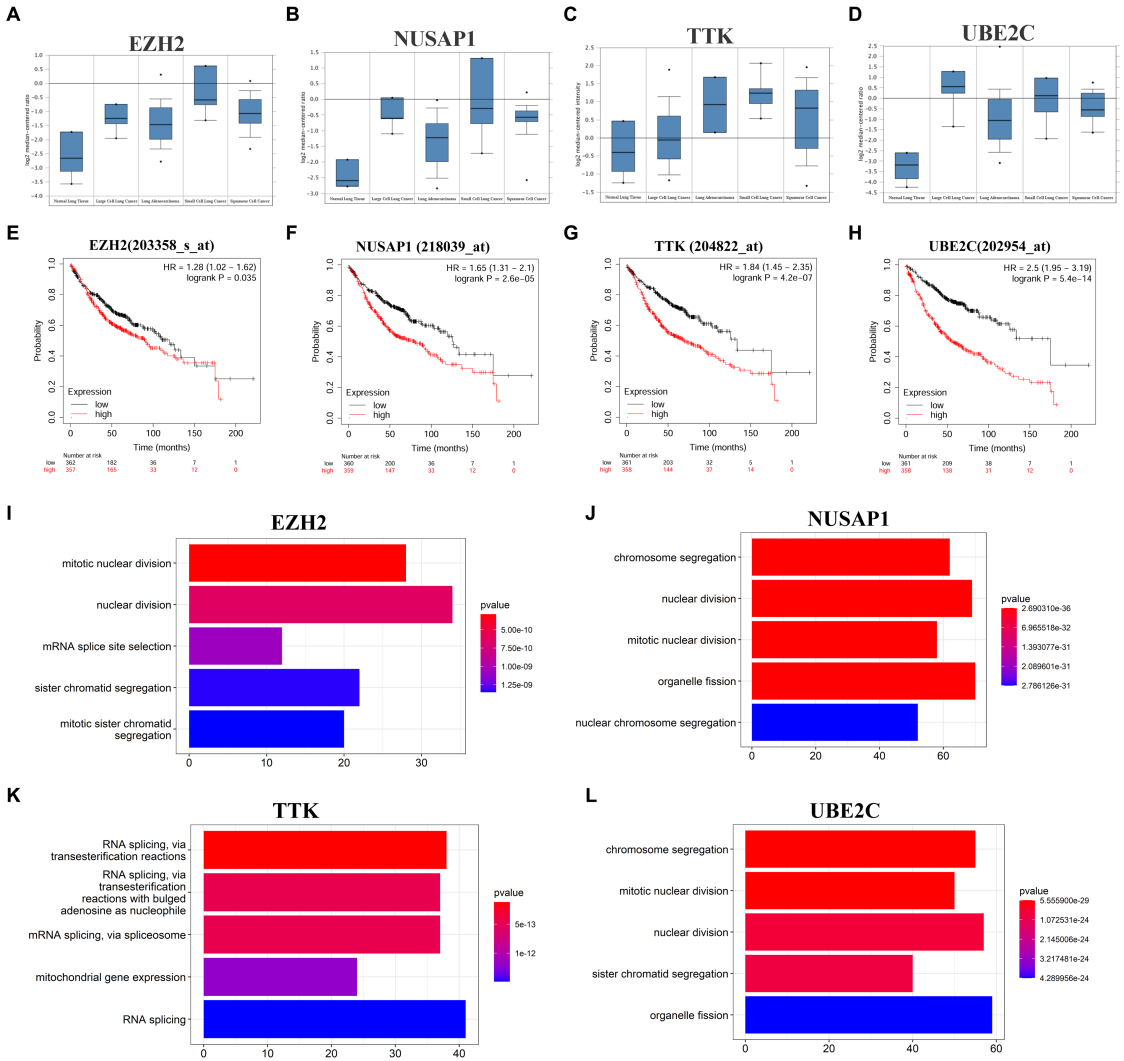
Four hub genes expression in different cancer types, prognosis in LUAD tissues and major cellular functions. **(A–D)** The expression of *EZH2*, *NUSAP1*, *TTK* and *UBE2C* in different tissues of normal, large cell lung cancer, LUAD, SCLC, and squamous cell lung cancer in the Garber lung tumor dataset. **(E–H)** The Kaplan–Meier plotter online platform was used for survival analysis of hub genes, and *p* < 0.05 was considered to be statistically significant. **(I–L)** Visualization of GO enrichment analysis of the first 500 positively related genes in the hub gene set based on the clusterProfiler package of the R project (enrichment results of the first 5 biological processes are displayed).

Cytoscape software identified 4 hub genes, which were all upregulated in SCLC tissues. Their names, abbreviations, and functions are shown in Table 1. The Oncomine database was used to analyze the expression of these hub genes in different normal, large cell lung cancer, LUAD, SCLC, and squamous cell lung cancer tissues (Figures 2A–D). There was no significant difference between *EZH2* in large cell lung cancer, LUAD, and squamous cell lung cancer, but its expression significantly increased in SCLC. *NUSAP1* expression in SCLC was significantly higher than that in LUAD, and the average level of SCLC sample data was higher than that of large cell lung cancer and squamous cell lung cancer; the average expression level of *TTK* in SCLC was higher than that of normal, large cell lung cancer, LUAD and squamous cell lung cancer. The average expression level of *UBE2C* was higher in large cell lung cancer and SCLC, while the expression level was lower in normal, LUAD, and squamous cell lung cancer tissues. This indicated that hub genes played an important role in the development from LUAD into SCLC, and their functional role in LUAD was not as strong as that in SCLC. Then, we used the Kaplan–Meier curve feature in the Kaplan–Meier plotter database to analyze survival-related hub genes, which included 719 LUAD patients (Figures 2E–H). We noticed that patients with elevated levels of *EZH2*, *NUSAP1*, *TTK*, and *UBE2C* were associated with a decrease in overall survival (*p* < 0.05).

**Table 1:** Functional roles of hub genes with a degree ≥8.

Gene symbol	Full name	Function
*EZH2*	Enhancer of zeste homolog 2	This gene is the functional enzymatic component of the polycomb repressive complex 2 (PRC2), which is responsible for healthy embryonic development through the epigenetic maintenance of genes responsible for regulating development and differentiation
*NUSAP1*	Nucleolar and spindle-associated protein 1	This gene is a nucleolar-spindle-associated protein that plays a role in spindle microtubule organization
*TTK*	Threonine and tyrosine kinase	This gene encodes a dual specificity protein kinase with the ability to phosphorylate tyrosine, serine and threonine. Associated with cell proliferation, this protein is essential for chromosome alignment at the centromere during mitosis and is required for centrosome duplication. It has been found to be a critical mitotic checkpoint protein for accurate segregation of chromosomes during mitosis
*UBE2C*	Ubiquitin-conjugating enzyme E2C	The modification of proteins with ubiquitin is an important cellular mechanism for targeting abnormal or short-lived proteins for degradation. This gene encodes a member of the E2 ubiquitin-conjugating enzyme family. The encoded protein is required for the destruction of mitotic cyclins and for cell cycle progression, and may be involved in cancer progression

To further analyze the potential mechanism of the influence of the hub gene set on SCLC, the co-expressed genes of the hub gene set in the SCLC transcriptome data were screened through the cBioProtal data analysis platform, and the Spearman correlation coefficient was above 0.3. Table 2 lists the top 10 related genes of each hub gene. Through the correlation between the Spearman correlation coefficient and gene expression, the clusterProfiler package of the R project was used to perform GO enrichment analysis on the first 500 positively related genes (Figures 2I–L). The results show that changes in *EZH2* expression mainly affect related functions such as mitotic nuclear division, nuclear division, mRNA splice site selection, sister chromatid segregation and mitotic sister chromatid segregation; changes in *NUSAP1* expression mainly affect chromosome segregation, nuclear division, mitotic nuclear division, organelle fission, nuclear chromosome segregation and other related functions; *TTK* expression changes mainly affect RNA splicing, mitochondrial gene expression and other related functions; *UBE2C* expression changes mainly affect chromosome segregation, mitotic nuclear division, nuclear division, sister chromatid segregation, organelle fission and other related functions, as shown in Supplementary Table S3. The enrichment analysis of related genes focused more on the cell cycle and was more inclined to the G2/M phase.

**Table 2:** cBioPortal analysis of the 10 genes most closely related to gene set.

Gene symbol	Correlated gene	Cytoband	Spearman’s correlation	*p*-value	*q*-value
EZH2	EML4	2p21	0.630965622	2.72E−10	2.25E−06
EZH2	RETSAT	2p11.2	−0.621522132	5.95E−10	2.25E−06
EZH2	GPR137C	14q22.1	0.620234869	6.61E−10	2.25E−06
EZH2	DPY19L2	12q14.2	0.619941283	6.77E−10	2.25E−06
EZH2	SCARB1	12q24.31	−0.619760614	6.87E−10	2.25E−06
EZH2	DTL	1q32.3	0.619083107	7.26E−10	2.25E−06
EZH2	SS18L1	20q13.33	0.616734417	8.77E−10	2.30E−06
EZH2	EFR3A	8q24.22	−0.615198735	9.91E−10	2.30E−06
EZH2	FAM111B	11q12.1	0.602880647	2.59E−09	5.35E−06
EZH2	CPSF4	7q22.1	0.594647696	4.82E−09	8.95E−06
NUSAP1	WDR76	15q15.3	0.785885276	3.65E−18	6.77E−14
NUSAP1	CCNB2	15q22.2	0.759372177	2.10E−16	1.95E−12
NUSAP1	ARHGAP11A	15q13.3	0.742773261	2.06E−15	1.27E−11
NUSAP1	ARPP19	15q21.2	0.727845528	1.39E−14	6.44E−11
NUSAP1	BUB1B	15q15.1	0.724570912	2.07E−14	7.69E−11
NUSAP1	CTDSPL2	15q15.3-q21.1	0.701897019	2.88E−13	8.92E−10
NUSAP1	ADAL	15q15.3	0.677416441	3.80E−12	1.01E−08
NUSAP1	OIP5	15q15.1	0.674119241	5.27E−12	1.22E−08
NUSAP1	UBE2C	20q13.12	0.66802168	9.58E−12	1.98E−08
NUSAP1	NDC80	18p11.32	0.664046974	1.40E−11	2.61E−08
TTK	TRMT11	6q22.32	0.763012438	1.24E−16	2.31E−12
TTK	LYRM2	6q15	0.730758808	9.65E−15	8.96E−11
TTK	RARS2	6q15	0.723125565	2.47E−14	1.53E−10
TTK	RNGTT	6q15	0.717908762	4.61E−14	1.90E−10
TTK	HDAC2	6q21	0.717028004	5.12E−14	1.90E−10
TTK	PPIL4	6q25.1	0.711472448	9.77E−14	3.03E−10
TTK	ZNF675	19p12	0.707971996	1.46E−13	3.71E−10
TTK	FBXO5	6q25.2	0.707158988	1.60E−13	3.71E−10
TTK	ORC3	6q15	0.705352304	1.96E−13	4.04E−10
TTK	ZNF93	19p12	0.700271003	3.45E−13	6.40E−10
UBE2C	CCNB2	15q22.2	0.761562782	1.53E−16	2.85E−12
UBE2C	BIRC5	17q25.3	0.753884372	4.56E−16	4.08E−12
UBE2C	CDCA5	11q13.1	0.751219512	6.59E−16	4.08E−12
UBE2C	UBE2T	1q32.1	0.724141825	2.18E−14	1.01E−10
UBE2C	RAE1	20q13.31	0.721883469	2.87E−14	1.07E−10
UBE2C	GADD45GIP1	19p13.13	0.707136405	1.60E−13	4.83E−10
UBE2C	ALYREF	17q25.3	0.706007227	1.82E−13	4.83E−10
UBE2C	SAC3D1	11q13.1	0.702597109	2.67E−13	5.94E−10
UBE2C	TUBA1B	12q13.12	0.701897019	2.88E−13	5.94E−10
UBE2C	KIF15	3p21.31	0.693902439	6.88E−13	1.28E−09

### 3.3. Changes of hub genes RNA expression in LUAD cell lines after RB1 knockdown

**Figure 3:**
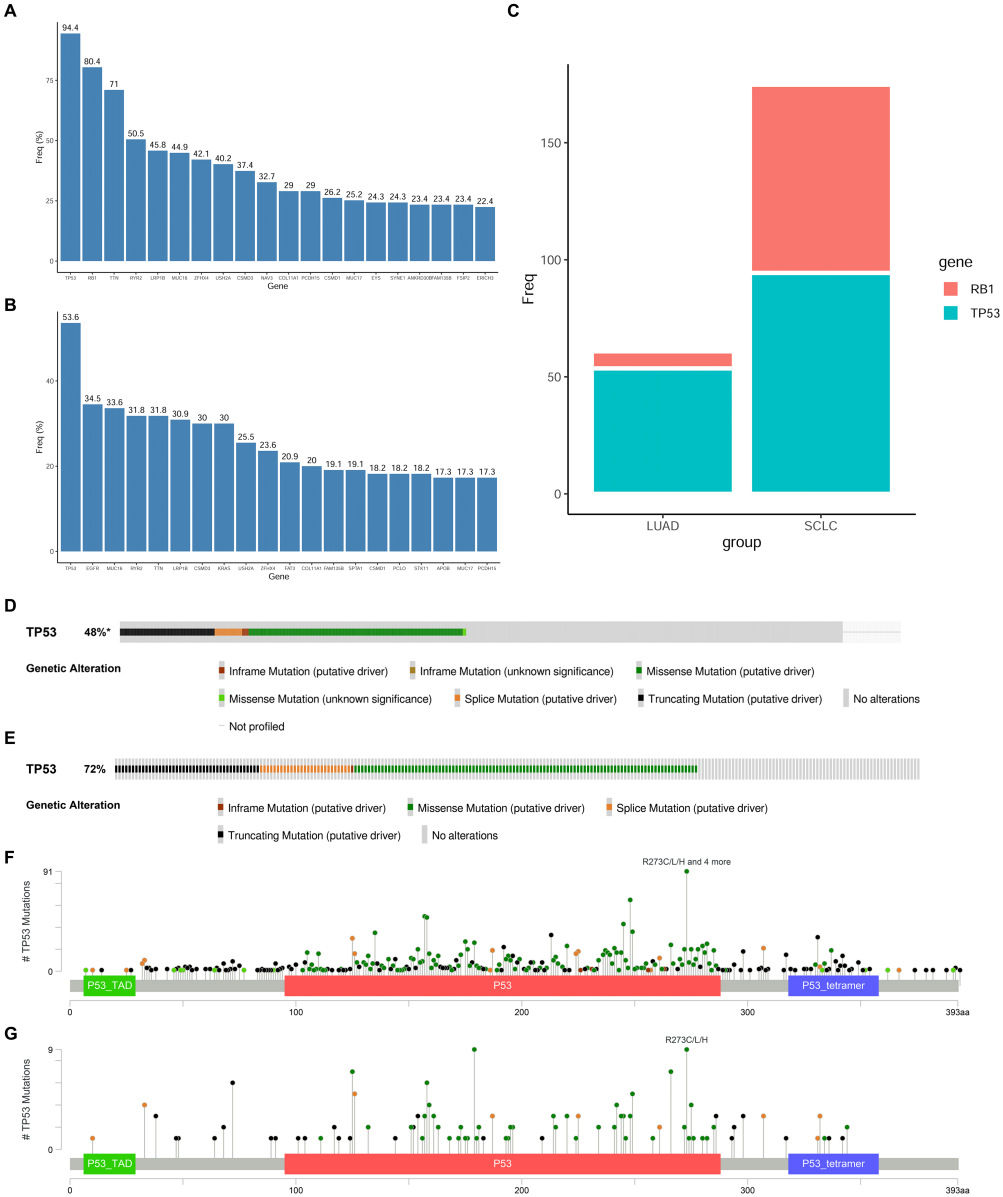
Mutation frequency of RB1 and TP53 in SCLC and LUAD tissues. **(A,B)** Top 20 hot spot mutation genes and their mutation frequencies in SCLC and LUAD derived from the cBioPortal website; **(C)** comparison of *RB1* and *TP53* mutation frequencies in LUAD and SCLC; **(D)** the main mutation types of TP53 in LUAD tissues; **(E)** the main mutation types of TP53 in SCLC tissues; **(F)** the main mutation sites of TP53 in LUAD tissues; **(G)** the main mutation sites of TP53 in SCLC tissues.

The genome of SCLC is characterized by high frequency mutations in TP53 and RB1, which are thought to be early events in transformation. We first obtained the gene mutation profiles of LUAD and SCLC from the cBioPortal website^6^ and compared the mutation frequencies of TP53 and RB1 mutations in these two tumors (Figures 3A–C). The double mutation of RB1 and TP53 is higher in SCLC. The important role of RB1 and TP53 co-mutation and inactivation in the transformation of LUAD has also been clearly reported. In LUAD and SCLC tissues, missense mutations, truncating mutations, and splicing mutations occupy the top three TP53 mutations, respectively, (Figures 3D,E), and TP53^R273C/L/H^ mutations are the most common mutation types of TP53 in LUAD and SCLC tissues (Figures 3F,G). To determine the changes in 4 core genes under the premise of inactivation mutations of RB1 and TP53 in the early process of NSCLC transformation into SCLC, we selected NCI-H1299 and NCI-H1975 lung adenocarcinoma cell lines. H1299 cells have TP53 protein deletion, and H1975 cells have EGFR^L858R/T790M^ and TP53^R273H^ mutations, in which TP53^R273H^ results in reduced activation of *TP53* target gene expression, and conferred resistance to EGFR-TKI drugs in cells with EGFR mutations. We knocked down RB1 in the above two cell lines, respectively, to construct a cell model in which TP53 and RB1 were co-inactivated.

**Figure 4:**
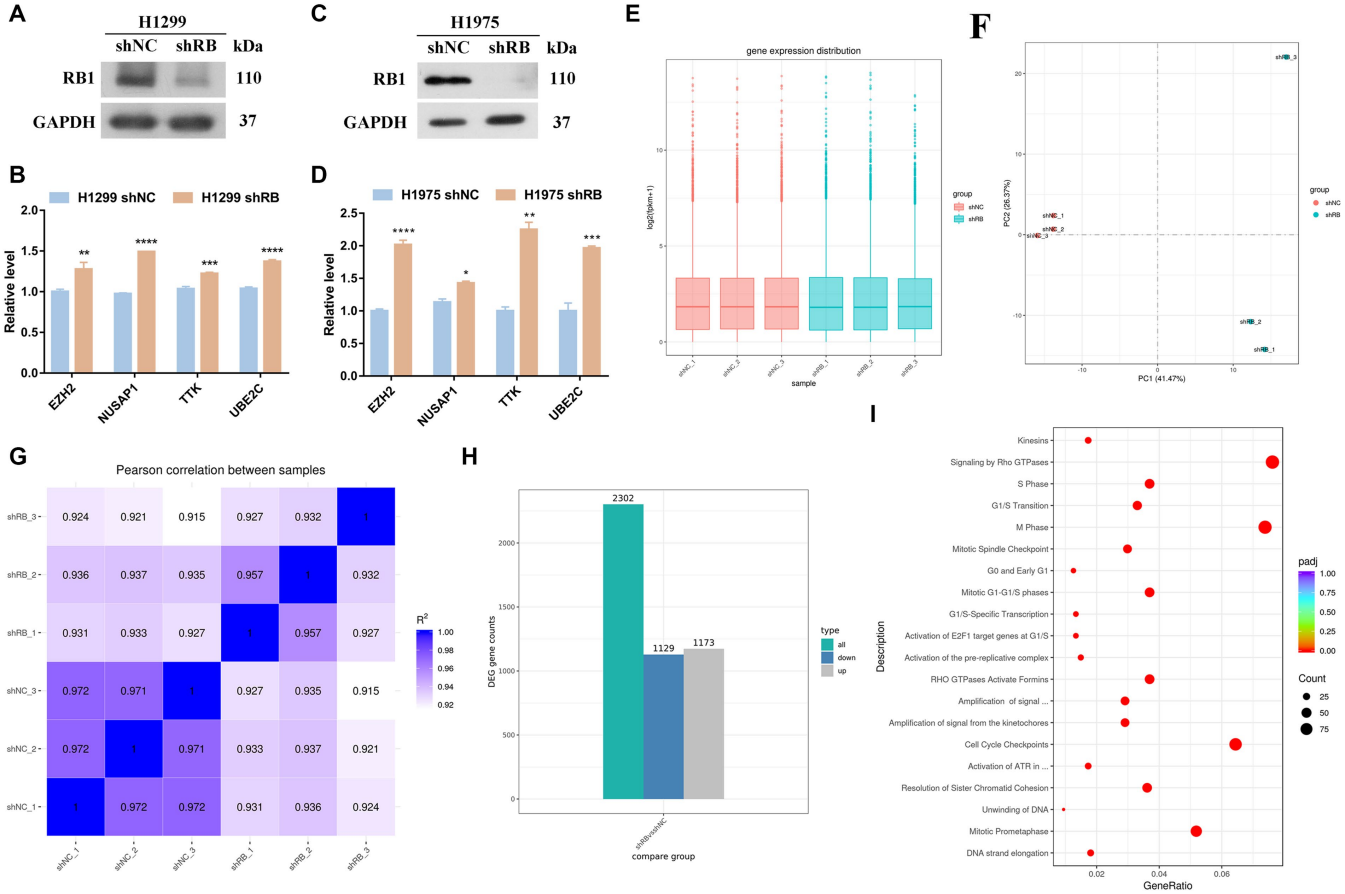
The mRNA expression changes of four hub genes in the LUAD cell line with RB1 knockdown. **(A)** WB was used to detect whether H1299 cells successfully knocked down RB1; **(B)** qPCR was used to detect the RNA expression changes of four hub genes in H1299 cells knocked down RB1; **(C)** WB was used to detect whether H1975 cells successfully knocked down RB1; **(D)** qPCR was used to detect the RNA expression changes of four hub genes in H1975 cells knocked down RB1; **(E)** box plots of expression levels under different experimental conditions; **(F)** shows the principal component analysis; **(G)** shows the Pearson correlation coefficient analysis; **(H)** shows the differentially expressed RNAs histogram, where 2,302 differentially expressed RNAs were identified, including 1,173 up-regulated RNAs and 1,129 down-regulated RNAs; **(I)** reactome pathway enrichment scatter plot.

As shown in Figures 4A–D, the RNA expressions of the four hub genes were all increased in the RB1-knockdown H1299 cells and H1975 cells (*p* < 0.05); The natural mutation state possessed by H1975 cells is the best choice and can reflect the state of LUAD cells before clinical transformation. H1975 cells was then sequenced (Supplementary Table S4). Figures 4E–G is the sample repeatability test, and the repeatability between samples is good overall; Figure 4H shows the qualitative results of RNA. Differentially expressed RNAs were screened using specific criteria, namely an absolute value of log2FoldChange greater than 0 and an adjusted *p*-value less than 0.05. A total of 2,302 differentially expressed RNAs were identified in this test, of which 1,173 RNAs were up-regulated and 1,129 RNAs were down-regulated. By enriching the reactome pathway of all differentially expressed genes, knockdown of RB1 was found to have the most significant effect on the cell cycle (Figure 4I).

### 3.4. Changes in hub gene protein expression in LUAD cell lines after RB1 knockdown

**Figure 5:**
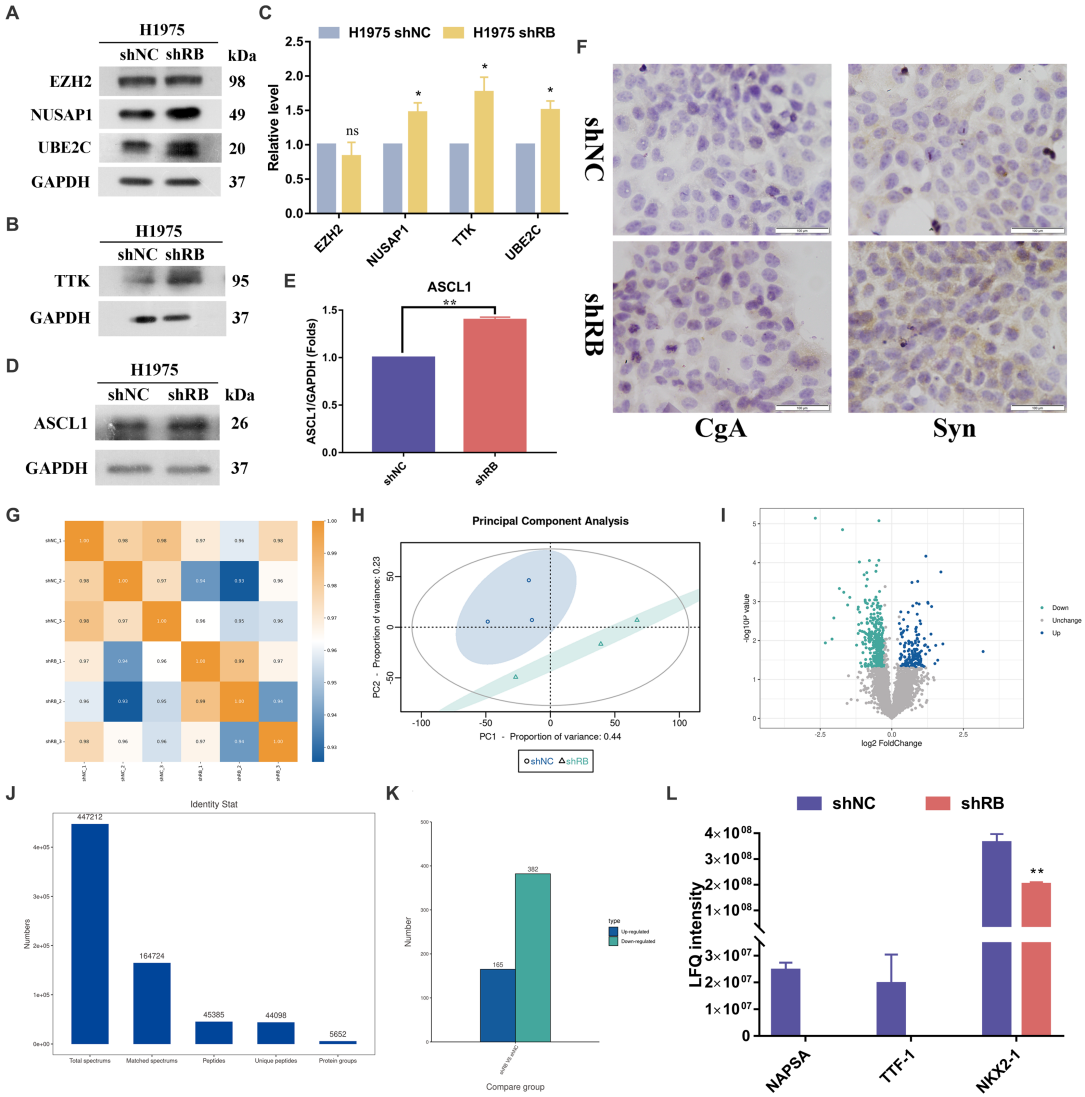
The protein expression changes of four hub genes in the H1975 cells with RB1 knockdown. **(A–C)** The expression of four hub proteins was compared between the H1975 shRB group and the H1975 shNC group. **(D–E)** shows the changes in ASCL1 protein expression in the constructed LUAD cell model using WB analysis. **(F)** Immunocytochemistry detection of the expression changes of NE markers (CgA, Syn) in H1975 cells after treatment. **(G)** Shows the Pearson correlation coefficient analysis. **(H)** Shows the principal component analysis. **(I)** The differential protein screening results are displayed in the form of a volcano plot. Blue for up-regulated proteins, cyan for down-regulated proteins, gray and black for no difference. **(J)** Displays the qualitative protein results, indicating the detection of a total of 5,652 proteins. **(K)** Shows the differentially expressed protein histogram, where 547 differentially expressed proteins were identified, including 165 up-regulated proteins and 382 down-regulated proteins. **(L)** NAPSA, TTF1 and NKX2-1 protein expression histogram.

We further detected the protein expression of hub genes in RB1 knockdown H1975 cells. Except for the expression of EZH2, which had no significant difference after *RB1* knockdown. The other three proteins (TTK, NUSAP1, UBE2C) were all increased after *RB1* knockdown, and the differences were statistically significant (Figures 5A–C). To further investigate whether the co-mutation and inactivation of RB1 and TP53 would lead to upregulation of ASCL1 which is a lineage transcription factor required for neuron and neuroendocrine differentiation and frequently high expression in approximately 75% of all SCLC subtypes (24). As shown in Figures 5D,E, the expression of ASCL1 in H1975 cells was significantly increased after knocking down RB1, which was statistically significant (*p* < 0.05).

We detected the mRNA expression of ASCL1, as shown in Figure S2A, after knocking down RB1, the expression of ASCL1 mRNA decreased. This result was contrary to the trend of ASCL1 protein expression. To explore the reason for the discrepancy between RNA and protein expression, we used MG132, a proteasome inhibitor that can effectively block the proteolytic activity of the 26S proteasome complex. After 15uM of DAPT was used to act on H1975 shNC cells and H1975 shRB cells for 7 days, 10uM MG132 was used to act on the above two cells for 0 h, 4 h, and 8 h, respectively, and then WB was used to detect the expression of ASCL1 protein. As shown in Supplementary Figure S2B, the expression of ASCL1 protein increased with the duration of MG132. In addition, the assembly of mutant TP53-E2F1 in a specific region of the ID4 promoter can control the expression of ID4, and ID4 can promote the degradation of ASCL1 (25, 26). We detected the expression of ID4 protein and found that after knocking down RB1, the expression of ID4 protein in H1975 cells decreased significantly (Supplementary Figures S2C,D), indicating that it reduced the degradation of ASCL1 protein.

To further validate if RB1 inactivation would lead to neuroendocrine phenotype transformation in the shRB cells, we conducted immunocytochemistry in shRB group *vs* shNC group with synaptophysin (Syn) and chromogranin A (CgA) antibodies, the two commonly clinical markers for SCLC. As shown in Figure 5F significantly increased expression of Syn was detected in the shRB cells, while the CgA expression was marginally detected.

Label-free proteomic detection was subsequently performed on the LUAD cell model (Supplementary file: Tables S5, S6). The resulting protein sequencing data is presented in Figures 5G–L, which includes Figures 5G–H indicating the repeatability of the samples. Figure 5I shows the volcano plot of the differential protein screening results and Figure 5J displaying the qualitative results. In total, 5,652 proteins were identified and subsequently screened for differentially expressed proteins using specific criteria, including a multiple of difference greater than 1.2 times and a *p*-value of *t*-test detection less than 0.05. Following this analysis, 547 differentially expressed proteins were identified, with 165 proteins up-regulated and 382 proteins down-regulated, as shown in Figure 5K. Among the differentially expressed proteins, we found that three markers commonly used clinically to identify LUAD (NAPSA, TTF1 and NKX2-1) were significantly down-regulated in H1975 cells after knocking down the RB1 gene (Figure 5L).

### 3.5. Changes of hub gene expression in H1975 LUAD cell line after ASCL1 overexpression

**Figure 6:**
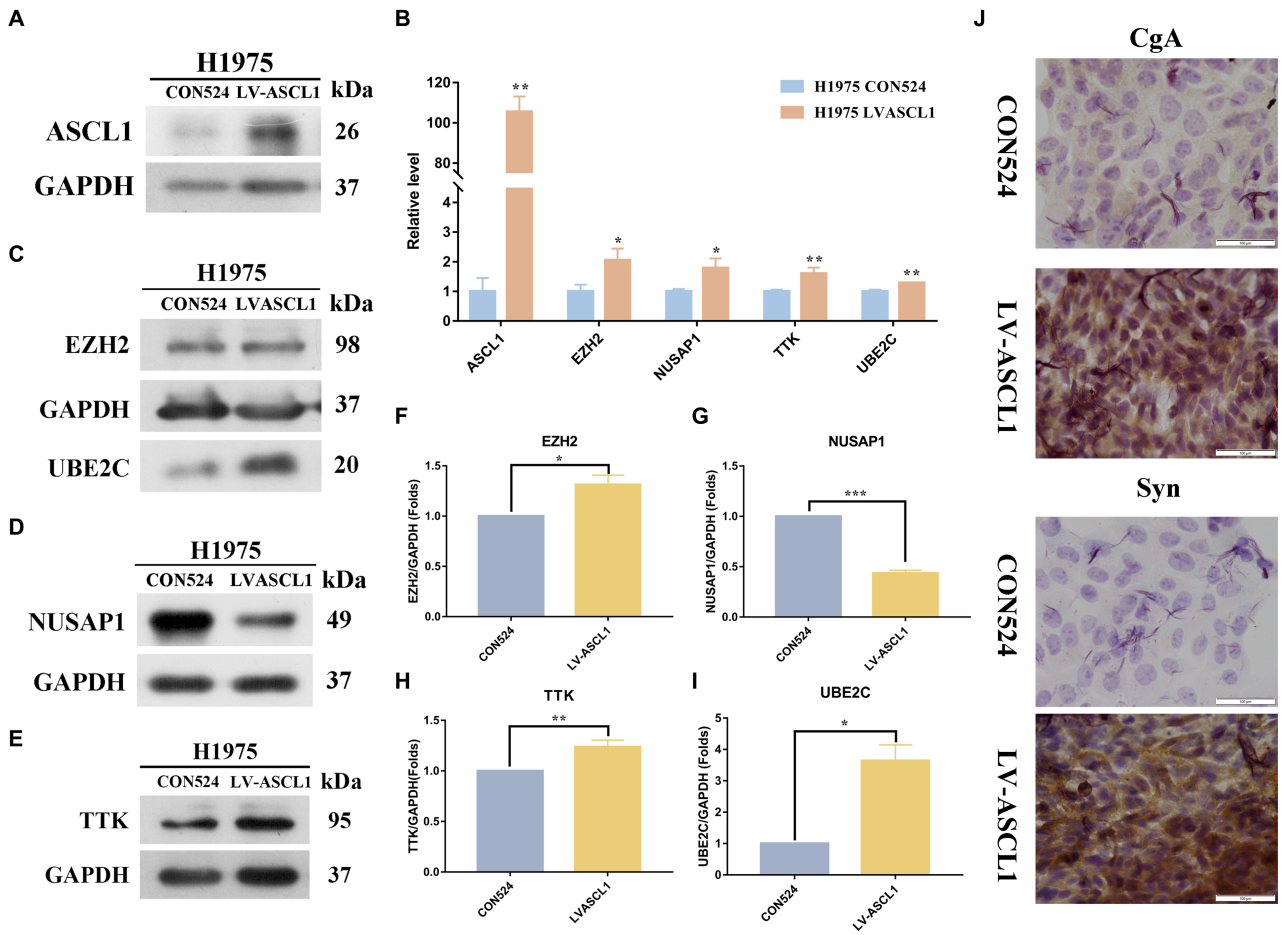
Effects of ASCL1 overexpression on hub gene expression and neuroendocrine markers in H1975 cells. **(A)** Constructed H1975 cells overexpressing ASCL1, and used WB to detect the expression of the target protein ASCL1; **(B)** qPCR was used to detect the RNA expression changes of four hub genes (*EZH2*, *NUSAP1*, *TTK*, *UBE2C*) in H1975 cells overexpressing ASCL1; **(C–I)** the expression of four hub proteins in H1975 cells overexpressing ASCL1 gene was detected by WB; **(J)** the expression changes of CgA and Syn in H1975 cells after ASCL1 overexpression were detected by immunocytochemistry.

Molecular typing dominated by the ASCL1 transcription factor accounts for about 75% of SCLC molecular typing, which is essential for the survival and growth of these cells (27, 28). In order to explore the changes in hub gene expression after ASCL1 protein expression increased, we first constructed H1975 cells overexpressing ASCL1, the control group was H1975 CON524, and the experimental group was H1975 LV-ASCL1. After construction, qPCR and WB were used to detect the expression of the target protein ASCL1, as shown in Figures 6A,B, after transfection with overexpressed lentiviral particles, the expression of ASCL1 protein in the experimental group was higher than that in the control group, suggesting that the H1975 cells overexpressing ASCL1 were successfully constructed.

Then we first detected the RNA expression changes of the hub gene by qPCR, as shown in Figure 6B. The results indicated that the RNA expressions of EZH2, NUSAP1, TTK, and UBE2C were all significantly increased. We further examined the protein expression of the hub gene (Figures 6C–I). The results showed that the expressions of three hub proteins EZH2, UBE2C, and TTK in H1975 cells were significantly increased, while the expression of NUSAP1 protein was decreased, and the difference was statistically significant (*p* < 0.05).

To detect the neuroendocrine transformation of cells, we detected the expressions of CgA and Syn in H1975 CON524 and H1975 LVASCL1 cells by immunocytochemistry. As shown in Figure 6J, after expressing ASCL1, the color development of CgA and Syn in the cytoplasm was enhanced, the distribution density increased, and the protein expression level increased.

### 3.6. Detection of TKIs drug sensitivity of treated H1975 cells

**Figure 7:**
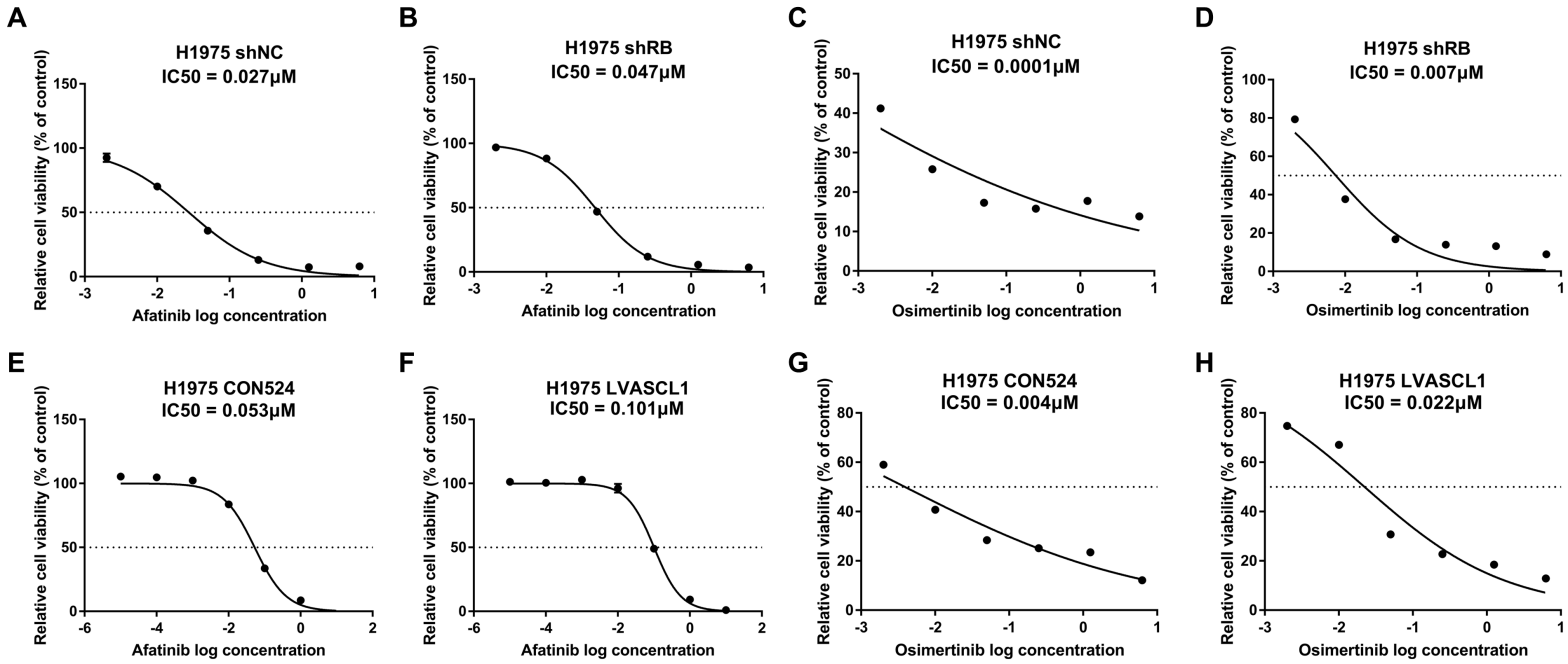
Detection of TKIs drug sensitivity of treated H1975 cells. **(A,B)** Detection of afatinib drug sensitivity in H1975 shNC and H1975 shRB cells; **(C,D)** detection of osimertinib drug sensitivity in H1975 shNC and H1975 shRB cells; (**E,F)** detection of afatinib drug sensitivity in H1975 CON524 and H1975 LVASCL1 cells; (**G,H)** detection of osimertinib drug sensitivity in H1975 CON524 and H1975 LVASCL1 cells.

In clinical practice, the transformation of LUAD into SCLC will lead to drug resistance to TKIs that are effective in the original treatment. We continued to examine whether knockdown of RB1 and overexpression of ASCL1 would lead to resistance of LUAD cells to TKIs. As shown in Figure 7, after knocking down RB1 or overexpressing ASCL1, the drug sensitivity of H1975 cells to afatinib and osimertinib decreased.

## 4. Discussion

Lung cancer is the leading cause of cancer deaths in the world. Although overall survival rates have increased in the past few decades, its morbidity and mortality are still among the highest of all cancers. Lung cancer can be divided into two categories, SCLC and NSCLC. SCLC is a highly aggressive tumor. Because SCLC originates from neuroendocrine cell precursors, it expresses neuroendocrine markers such as Syn and CgA, therefore, it is classified as a type of pulmonary neuroendocrine tumor (29). For LUAD patients with *EGFR* mutations, EGFR-TKI therapy is now a standard treatment option, but a small number of LUAD patients experience phenotype changes after EGFR-TKI treatment; that is, the pathological type of the tumor changes from LUAD to SCLC, and the tumor progresses rapidly leading to death. Transformed SCLC is usually resistant to EGFR-TKI treatment, the combination of platinum and etoposide was shown to be an effective treatment for transformed SCLC (30). However, the mechanism by which LUAD develops into SCLC remains unclear. Exploring the mechanism of transformation and looking for factors that promote transformation is urgently needed to improve the treatment and prognosis of these patients.

Herein, a series of bioinformatics analyses were performed on four independent gene chip databases (from LUAD and SCLC tissues), and 41 common DEGs were identified, of which 13 genes were upregulated and 26 genes were downregulated. Among the DEGs, four potential hub genes (*EZH2*, *NUSAP1*, *TTK* and *UBE2C*) were obtained using Cytoscape’s CytoHubba plug-in. Finally, by constructing RB1 and TP53 co-inactivation cell model and ASCL1 overexpressed LUAD cell model, they were confirmed that the expression of 4 genes, *EZH2*, *NUSAP1*, *TTK*, and *UBE2C* was significantly changed.

EZH2 is the enzyme subunit of polycomb repressive complex 2 (PRC2), which can catalyze the trimethylation of histone H3 at Lys 27 (H3K27me3) to regulate gene expression through epigenetic mechanisms (31). In addition, EZH2 inhibits differentiation by inhibiting lineage-specific factors (32–34); at the same time, it may also promote the differentiation of multiple tissue types by inhibiting related transcription programs (35–37). NUSAP1 is a microtubule and chromatin binding protein that cross-links microtubules during mitosis (38). The level of NUSAP protein expression is strictly regulated by the anaphase-promoting complex/cyclosome during the cell cycle (39, 40), and the expression of *NUSAP1* can be upregulated by the loss of RB1 on the RB1/E2F1 axis (41, 42). *TTK* was cloned and identified as a dual-specificity protein kinase that can phosphorylate tyrosine or serine/threonine residues (43). It shows the greatest activity in M phase (44). In addition, Liu et al. (45) observed that TTK is strongly expressed in human fetal liver but not in normal adult liver, and it is believed that TTK may promote carcinogenesis and tumor progression through dedifferentiation of hepatocytes into an embryonic state. UBE2C is a specific ubiquitin conjugating enzyme of the anaphase-promoting complex or cyclosome (APC/C) (46), which participates in the cell cycle process and checkpoint control by targeting the degradation of short-lived proteins. Cells overexpressing UBE2C ignore the mitotic spindle checkpoint signal and lose genome stability (47).

We found that upregulation of the four hub genes was associated with poorer overall survival, and most of them were significantly elevated in SCLC tissues. In order to explore the mechanism by which hub genes affect SCLC transformation, we used GO enrichment analysis to find that, in addition to the above functions, the expression of hub genes mainly affects the cell cycle, particularly the alterations in the G2/M phase. This alteration is related to the hub genes, but it is also consistent with the concurrent changes brought on by the biallelic deletions of *TP53* and *RB1*. However, neither *TP53* nor *RB1* are therapeutically targeted. In addition to proving that EZH2 inhibitors may stop lineage transition in experimental models (48), there are also drugs available that target the subsequent loopholes in the formation of the cell cycle. The first is an inhibitor of CHK1 and PLK1 that targets DNA damage checkpoints (49); the second is an inhibitor of AURK that regulates mitotic spindle assembly and chromosome segregation (50). All of these medicines provide more opportunities for patients with SCLC who are at risk or who have undergone transformation.

In this study, LUAD cell models with EGFR mutation, TP53 and RB1 co-mutation/inactivation, and ASCL1 overexpression were constructed to represent two different stages of transformation of LUAD into SCLC, the first one is the currently recognized prerequisite for transformation, that is co-mutation/inactivation of TP53 and RB1. At this stage, we found that the expression of lineage markers (NAPSA, TTF1, NKX2-1) (51–54) in LUAD cells was significantly reduced, and they were in the intermediate stage of transformation. The second is the increased expression of ASCL1, ASCL1 is a major regulator that induces neuronal and neuroendocrine differentiation, and ASCL1-positive SCLC expresses a full set of neuroendocrine markers (28, 55). In this study, the mRNA expression of four hub genes was significantly increased in both RB1 and TP53-inactivated LUAD cell lines and ASCL1-overexpressed LUAD cell lines, these results were consistent with the bioinformatics results based on transcriptome data, although protein expression was not necessarily the same.

In cytotoxicity experiments, whether RB1 was knocked down or ASCL1 was overexpressed, H1975 lung adenocarcinoma cells showed decreased drug sensitivity to TKIs, it shows that in TP53/RB1 co-mutated/inactivated LUAD cells, TKIs drug resistance begins, and TKIs drug resistance may further develop after neuroendocrine transformation. These results suggest that it is very necessary to actively switch treatment methods for LUAD patients with high risk of transformation (TP53/RB1 co-mutation/inactivation has occurred).

In this study, by screening multiple databases comparing LUAD and SCLC, *EZH2*, *NUSAP1*, *TTK* and *UBE2C* are believed to be the core genes that affect the transformation of LUAD to SCLC. This gene set is closely related to cell cycle regulation, posttranscriptional regulation, and DNA methylation. In addition, because this gene set is closely related to the occurrence of SCLC, it may have important value for the early diagnosis and treatment of SCLC transformation.

## Data availability statement

The original contributions presented in the study are included in the article/Supplementary material. The RNA sequencing data provided in the research are stored in the GEO repository, and the GEO accession number is GSE232387; The mass spectrometry proteomics data have been deposited to the ProteomeXchange Consortium via the PRIDE (56) partner repository with the dataset identifier PXD042073. Further inquiries can be directed to the corresponding author.

## Author contributions

YZ, QC, and YL designed the study. YZ collected the literature. YZ and QC designed and performed experiments. TH, DZ, and YZ performed statistical analyses. QC, TH, DZ, and YZ analyzed the data. YZ and YL wrote the manuscript. All authors contributed to the article and approved the submitted version.

## Conflict of interest

The authors declare that the research was conducted in the absence of any commercial or financial relationships that could be construed as a potential conflict of interest.

## Publisher’s note

All claims expressed in this article are solely those of the authors and do not necessarily represent those of their affiliated organizations, or those of the publisher, the editors and the reviewers. Any product that may be evaluated in this article, or claim that may be made by its manufacturer, is not guaranteed or endorsed by the publisher.

## Footnotes

^1^ http://www.ncbi.nlm.nih.gov/geo

^2^ https://www.ncbi.nlm.nih.gov/geo/geo2r/

^3^ http://bioinformatics.psb.ugent.be/webtools/Venn/

^4^ http://www.cbioportal

^5^ https://www.oncomine.org/resource/login.html

^6^ https://www.cbioportal.org/
