# Supplementary material for: Bioinformatics-based screening of key genes for transformation of tyrosine kinase inhibitor-resistant lung adenocarcinoma to small cell lung cancer
Source: Front Med (Lausanne). 2023 Jul 31;10:1203461. doi: 10.3389/fmed.2023.1203461 (PMC10424445; doi:10.3389/fmed.2023.1203461)
Supplement: Supplementary file 7 [file Image_1.pdf]

Supplementary Material

1 Supplementary Figures

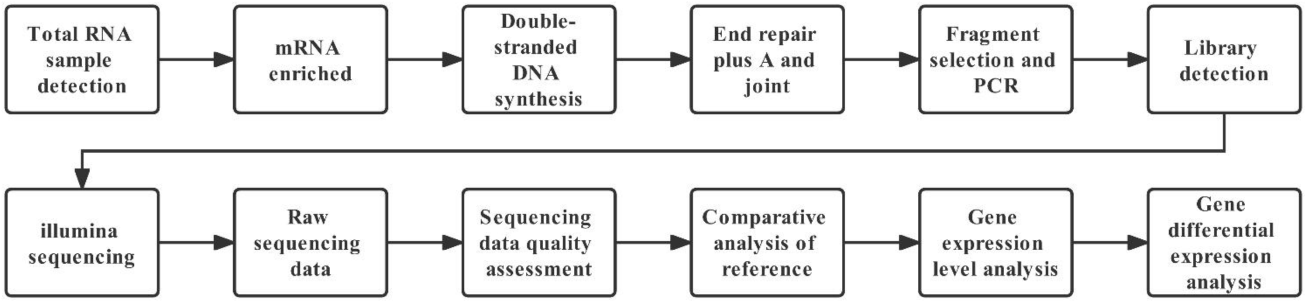

Supplementary Figure S1. Procedure of RNA-seq data analysis.

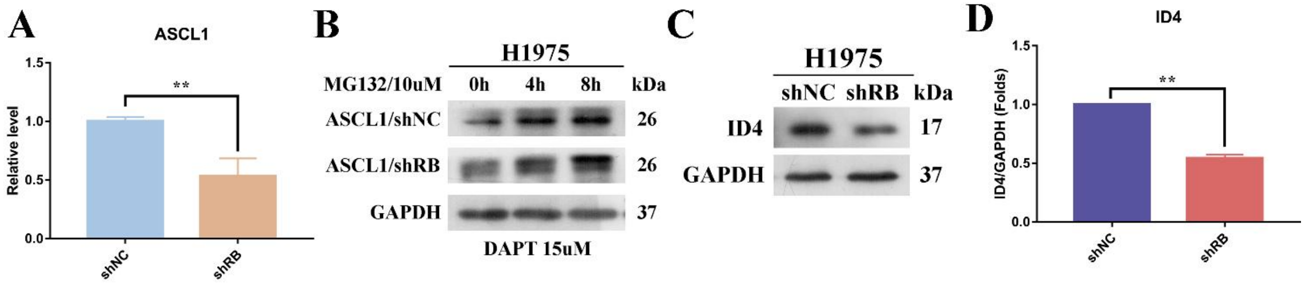

Supplementary Figure S2. Changes of ASCL1 mRNA expression and protein degradation level in RB1 knockdown H1975 cells. **A** The expression of ASCL1 mRNA in RB1-knockdown H1975 cells by qPCR. **B** The effect of proteasome inhibitor MG132 on the expression of ASCL1 protein. **C-D** The expression of ID4 protein in cells of shNC group and shRB group was detected by WB.
